# Antibacterial Activity of Ethanolic Extract of Cinnamon Bark, Honey, and Their Combination Effects against Acne-Causing Bacteria

**DOI:** 10.3390/scipharm85020019

**Published:** 2017-04-11

**Authors:** Elin Julianti, Kasturi K. Rajah, Irda Fidrianny

**Affiliations:** School of Pharmacy, Bandung Institute of Technology, Labtek VII, JL. Ganesha 10, Bandung 40132, West Java, Indonesia; kasturi_mas@yahoo.com (K.K.R.); irda@fa.itb.ac.id (I.F.)

**Keywords:** antibacterial activity, cinnamon, honey, checkerboards method, additive activity

## Abstract

*Propionibacterium acnes* and *Staphylococcus epidermidis* are the major skin bacteria that cause the formation of acne. The present study was conducted to investigate antibacterial activity of ethanolic extract of cinnamon bark, honey, and their combination against acne bacteria. The antibacterial activity of extract of cinnamon bark and honey were investigated against *P. acnes* and *S. epidermidis* using disc diffusion. Minimum inhibitory concentrations (MICs) and minimum bactericidal concentrations (MBCs) were attained using Clinical and Laboratory Standard Institute (CLSI) methods. The interaction between cinnamon bark extract and honey was determined using a checkerboards method. The results showed that the MICs of cinnamon bark extract and honey against *P. acne* were 256 µg/mL and 50% *v*/*v*, respectively, while those against *S. epidermidis* were 1024 µg/mL and 50% *v*/*v*, respectively. The MBC of cinnamon bark extract against *P. acnes* and *S. epidermidis* were more than 2048 µg/mL, whereas the MBC for honey against *P. acnes* and *S. epidermidis* were 100%. The combination of cinnamon bark extract and honey against *P. acnes* and *S. epidermidis* showed additive activity with a fractional inhibitory concentration index (FICI) value of 0.625. Therefore, the combination of cinnamon bark extract and honey has potential activity against acne-causing bacteria.

## 1. Introduction

Acne vulgaris is one of the most common skin disorders and mainly affects adolescents: around 40% of all 14–17-year old girls and 35% of all 16–19-year old boys [1]. Acne, by definition, is a multifactorial chronic inflammatory disease of pilosebaceous units that affects the skin of the face, neck, and upper trunk. Acne develops when these specialized follicles undergo pathologic alteration that results in the formation of non-inflammatory lesions (comedons) and inflammatory lesions (papules, pustules, and nodules). In general, *Staphylococcus epidermidis* and *Propionibacterium acnes* are the major skin bacteria that cause this formation of acne. Topical as well as systemic therapies are available for acne treatments. A topical therapy usually includes comedolytic agents, antibiotics, and various anti-inflammatory drugs, whereas systemic therapy covers antibiotics, zinc, and hormones for acne treatments. However, an excessive use of antibiotics over a long period can lead to the increasing resistance of acne bacteria. To overcome antibiotic resistance, essential oils and medicinal plant extracts present alternative solutions that are safer, more efficacious, and multifunctional. In topical acne treatments, medicinal plant extracts have fewer side effects than synthetic agents [2].

In some research reports, cinnamon has shown potential activity against acne bacteria. Cinnamaldehyde, a major constituent of cinnamon, shows anti-inflammatory activity. It inhibits the production of nitric oxide, which is responsible for inflammatory conditions in the human body. Moreover, cinnamon has also been shown to prevent the production of COX-2, a pro-inflammatory agent. Therefore, cinnamon has antibacterial and anti-inflammatory properties. The chemistry of cinnamon bark oil primary contains a cinnamaldehyde that is different from that found in cinnamon leaf oil (eugenol) and root-bark oil (camphor) [3].

Similar to cinnamon’s biological activity, honey works as a natural antibiotic, killing bacteria that cause acne. The anti-inflammatory properties of honey reduce the redness of acne. Its acidic property does not allow the bacteria to grow [4]. Honey releases hydrogen peroxide, which is an antibiotic that can also remove bacteria and clear acne [5]. Moreover, honey contains natural antioxidants, which can scavenge free radicals [6].

This paper investigates the antibacterial activity of an ethanolic extract of cinnamon bark, honey, and their combination against acne-causing bacteria. The antibacterial activity of cinnamon bark extract and honey were investigated against *P. acnes* and *S. epidermidis* using disc diffusion. Minimum inhibitory concentrations (MICs) and minimal bactericidal concentrations (MBCs) were attained using Clinical and Laboratory Standard Institute (CLSI) methods.

## 2. Materials and Methods

### 2.1. Materials

Cinnamon bark and forest honey were obtained from Indonesia. The reflux apparatus and other chemicals were of analytical grade. Test bacteria were *Propionibacterium acnes* and *Staphylococcus epidermidis* obtained from culture collections of the Microbiology Laboratory, School of Pharmacy, Bandung Institute of Technology, Bandung, Indonesia.

### 2.2. Sample Preparation

Firstly, cinnamon bark was washed and cut into small pieces. Cinnamon bark was dried using a drying cabinet at 40–42 °C for 3–4 days. The dried plant material was ground into powder.

### 2.3. Preparation of Ethanolic Extract

As much as 300 g of cinnamon bark crude drug (the powder of cinnamon bark) was weighed and placed into a flask. Ethanol solvent with a concentration of 96% was added to the flask until the plant materials were submerged by the solvent. The extraction process took 2 h after boiling the solvent and was repeated three times. The ethanol extract was combined and evaporated using a rotary evaporator.

### 2.4. Characterization of Crude Drug and Extract

Cinnamon bark crude drug was characterized by microscopy for the identification of drugs on a cellular level and continued by other characterizations, such as water content determination and phytochemical screening, for quality control of the crude drugs. Characterization of cinnamon bark extract included water content, total ash, ethanol, water-soluble extractable matter, and phytochemical screening. Methods for crude drug and extract characterization were adopted from Materia Medika Indonesia [7] and Biological and Phytochemical Screening of Plants [8].

### 2.5. Determination of Honey Quality

The sweet honey quality test was performed for the honey parameters of the National Standardization Agency 2004, including the hydroxymethylfurfural (HMF) content, diastase enzyme activity, moisture content, the level of sucrose, glucose level, ash content, water-insoluble solids, and metal contamination [9].

### 2.6. Antibiotic and Extract Preparation

Extracts of cinnamon bark and tetracycline HCl were dissolved in 1 mL of 100% DMSO prepared as a stock solution. Then, 0.1 mL of the stock solution was taken and diluted with 0.9 mL of Muller–Hinton broth (MHB), and the total volume of the solution was 1 mL. The tetracycline was used as a positive control.

### 2.7. Preparation of Bacterial Suspension

The agar slant was scratched using an ose needle with the microbe of interest, and kept in an incubator at 35 ± 2 °C for 24 h. On the following day, the microbe that had grown on the surface of the agar slant was slowly removed using the ose needle again and suspended in the MHB and incubated for 18–24 h at 35 ± 2 °C. The next day, the suspended microbe was diluted to attain an absorbance between 0.08 and 0.13 (0.5 McFarland). After it fulfilled the absorbance requirement, the 0.5 McFarland suspensions were diluted again with MHB, 1 mL bacterial suspension was added with 20 mL MHB, with a result of 5 × 10^6^ colony forming units (CFU)/mL [10].

### 2.8. Determination of Total Colony Forming Unit

The microbe was grown on a Petri dish by mixing 1 mL of bacterial suspension homogeneously with 15–20 mL of nutrient agar (NA) for several dilutions. Then, the agar was incubated for 24 h, and the colony forming unit was calculated using Colony Counter equipment. The concentration of the initial bacterial suspension (stock) was calculated from the Petri dish containing 30–300 CFU [10].

### 2.9. Antibacterial Screening

Antibacterial screening was performed by disc diffusion. Briefly 20 mL of NA was plated in a Petri dish with 1 mL of bacterial suspension. The sterilized paper discs (6 mm in diameter) were placed on solidified agar plates and inoculated with 10 µL of cinnamon bark extract, honey, and tetracycline HCl at various concentrations. Ethanol solvent with a concentration of 96% was used to dissolve the extract, and distilled water used to dissolve honey. A blank paper disc with 10 µL of 96% ethanol solvent was used as a negative control. Activity was determined after 24 h of incubation at 37 °C. The zone of inhibition was measured using Vernier’s caliper. Each microbe was prepared in two replications, and the average of inhibition zones was taken [10].

### 2.10. Determination of Minimum Inhibitory Concentration

The MICs of the cinnamon bark extract and honey against bacteria was determined with the microdilution method. This method was performed using a 96-well microplate, which consists of 12 columns and 8 rows. Initially, all wells were supplemented with 100 µL of MHB, except for Column 12, which was supplemented with 200 µL of MHB and used as a sterility control. Column 11 was supplemented with 100 µL of bacterial suspension and used as a growth control. As much as 100 µL of the cinnamon bark extract was added to Column 1; then, after diluting steps were taken, 100 µL from Well Column 1 was transferred to Well Column 2 with a micropipette. After that, 100 µL from Well Column 2 was transferred to Well Column 3. This step was repeated until Well Column 10 was reached. Then, the same steps were conducted for honey and tetracycline HCl (as a positive control). Finally, 100 µL of bacterial suspension was added to Columns 1–10. The microdilution plate was incubated at 35 ± 2 °C for 18–24 h [10].

### 2.11. Determination of Minimum Bacterial Concentration

A total of 15 mL of Muller–Hinton agar (MHA) was poured into a Petri dish and was left to solidify. The MBC of samples was determined following the MIC assay. From all wells that exhibited no apparent bacterial growth, the samples were streaked on the surface of the agar with an ose needle. After that, the Petri dish was incubated at 35 ± 2 °C for 18–24 h. The lowest concentration, which did not grow on the subculture, was recorded as the MBC.

### 2.12. Determination of Fractional Inhibitory Concentration Index

The antibacterial activity combination of cinnamon bark extract and honey against *Propionibacterium acnes* and *Staphylococcus epidermidis* was evaluated by determining the fractional inhibitory concentration index (FICI) value using the microdilution checkerboard method [10]. Firstly, 100 µL of MHB was added to all the wells. Then, 100 µL of cinnamon bark extract with four times the MIC value was added to the first row. Thereafter, 100 µL from the first row was transferred to the second row, 100 µL from the second row was transferred to the third row, and so on until Row 7 was reached. Next, 100 µL of pure honey (100%) was added to the first column. Then, 100 µL from the first column was transferred to the second column and so on until Column 9 reached. After that, 100 µL of the bacterial suspension was added to each well. Finally, the microplate was incubated at 35 ± 2 °C for 18–24 h. The concentration of the individual compounds in the combination of cinnamon bark extract and honey, which prevented visible growth, was recorded as the MIC of the individual compounds in the respective combination. The FICI for the combination was calculated as followed: FICI = FIC of Drug A + FIC of Drug B, where FIC A is the MIC of Drug A in the combination/MIC of Drug A alone, and FIC B is the MIC of Drug B in the combination/MIC of Drug B alone. The combination is considered synergistic when the FICI is ≤0.5, indifferent/additive when the FICI is >0.5 to <2, and antagonistic when the FICI is ≥2 [11].

## 3. Results and Discussions

**Figure 1:**
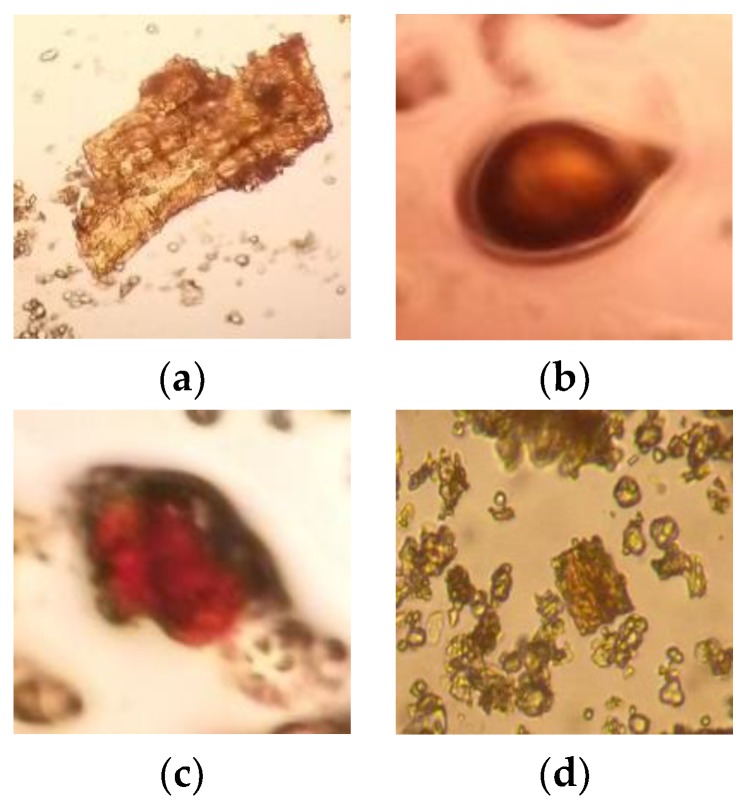
Microscopic characterization of *Cinnamomum* sp. bark powder, magnification 100×: (**a**) Parenchyme cells with starch; (**b**) Resin cell; (**c**) Cells with reddish brown contents; (**d**) Crystal bearing cell.

Cinnamon bark was bought from an herbal medicine store in Bandung, Indonesia. Firstly, the bark was selected to ensure that the bark used was not damaged and was free from foreign matter. Then, the selected bark was washed about three times using clean and fast flowing tap water to remove soil and other contaminants. The bark was then cut into small pieces to increase the surface area and to minimize the drying process period. After that, the drying process was conducted in a drying cabinet at 40–42 °C for 3–4 days. The drying process was essential to reduce the water content present in the crude drug, in which water can promote the growth of microorganisms such as yeast and molds. After the drying process was completed, the crude drug was ground into powder. The purpose was to minimize the crude drug particle size, which can increase the surface of the contact area, which makes the extraction process easier. Then, the crude drug was stored in a closed container and protected from light. The microscopic characterization of *Cinnamomum* sp. bark powder is shown in Figure 1.

The powdered crude drug was extracted with the reflux method. About 300 g of crude drug was dissolved in 1 L of 96% ethanol as the solvent system. The extraction process was repeated three times to obtain an optimum yield. The solvent was then removed using a rotary evaporator to obtain the concentrated extract. Then, characterization of the extract and crude drug was conducted by determining water content, density, total ash, water- and ethanol-soluble extractable matter, and phytochemical screening. Characterization extract and crude drug are important for ensuring the consistency of the plant extract in terms of quality, safety, and efficacy. The result of the characterization of crude drug and extract are shown in Table 1 and Table 2.

Based on phytochemical screening (Table 2), the crude drug and ethanolic extract of cinnamon bark contained alkaloid, flavonoid, steroid/triterpenoid, tannin, and quinone, and there was an absence of saponin. Results were similar to a previous study showing that *Cinnamomum cassia* bark extract contained phenols, alkaloids, steroids, and tannins, while saponins and glycosides were not detected in the test extract [12].

Furthermore, there are several methods for determining the characteristics of plant extracts. Firstly, the water content was determined by azeotropic distillation. This method was selected to determine the water content of the cinnamon bark crude drug because it contains a volatile compound that, can show inaccurate results when determining water by a gravimetric method. Based on the evaluation, the water content of cinnamon bark crude drug powder was 9.0% (*v*/*w*), and the water content of ethanolic cinnamon bark extract was 7.0% (*v*/*w*).

An amount of 1 kg of cinnamon bark after grinding was 950 g, so the percentage of the yield was 95% (*w*/*w*). Three hundred grams of cinnamon bark powder was extracted by the reflux method and concentrated using a rotary evaporator. The final weight of the extract was 47.67 g, so the yield percentage of the extract was 15.93% (*w*/*w*).

The quality test of honey for parameters such as HMF, diastase enzyme activity, moisture content, level of sucrose, glucose level, ash content, water-insoluble solids, and metal contamination including lead and copper fulfilled the requirements based on Standard National Agency of Indonesia.

In this experiment, two tested bacteria strains were chosen: *Propionibacterium acnes* and *Staphylococcus epidermidis*, obtained from the Microbiology Laboratory, School of Pharmacy-Bandung Institute of Technology. The bacterium cultures were streaked on a solidified slant nutrient agar and incubated for 24 h at 37 °C. After 24 h of growth, the bacteria were inoculated in nutrient broth (NB) and incubated again for 24 h. The suspension of bacteria was prepared at absorbance ranges between 0.08 and 0.13 using a UV-Vis spectrophotometer at a wavelength of 625 nm. Based on the determination of total CFU, a serial dilution procedure for each bacterium to 10^7^ and 10^8^ was performed afterward. The result for *P. acnes* at an absorbance of 0.093 was 154 × 10^7^ and 146 × 10^8^, whereas that for *S*. *epidermidis* at an absorbance of 0.083 was 271 × 10^7^ and 177 × 10^8^, and both bacteria strains fulfilled the CFU normal range of 30–300.

The antibacterial screening was performed by disc diffusion to determine the inhibition zone of the cinnamon bark extract, honey, tetracycline HCl, and the solvent of 96% ethanol against *P. acnes* and *S. epidermidis*. The result of the antibacterial screening of cinnamon bark extract, honey, and tetracycline HCl 250 µg/mL is presented in Table 3. The highest activity of cinnamon bark extract was 17.2 mm of the inhibition zone diameter found against *P. acnes*, followed by 16.8 mm against *S. epidermidis*, whereas the control disc used for the solvent (96% ethanol) had no zone of inhibition. Furthermore, the activity of honey was 16.2 mm of the inhibition zone diameter observed against *P. acnes*, while *S. epidermidis* was 16.7 mm. The reference standard tetracycline at 250 µg/mL had an inhibition zone diameter against *P. acnes* of 18.9 mm; against *S. epidermidis*, 24.8 mm. Based on the inhibition zone diameter of cinnamon bark extract and honey against bacteria more than 16 mm, it can be concluded that cinnamon bark extract and honey can be categorized as potent against both bacteria.

Cinnamon bark extract was shown to have a better inhibitory effect against *P. acnes* with a MIC of 256 µg/mL, followed by *S. epidermidis* with a MIC of 1024 µg/mL. Meanwhile, the honey was shown to have the same MIC against both *P. acnes* and *S. epidermidis*, which was 50% (*v*/*v*).

The MBC was obtained after determining the MICs. The MBC is the least amount of drug required to kill 99.9% of bacteria. The MBC of the sample test was determined by inoculating the sample from the well that showed no apparent bacterial growth. The samples were streaked with an ose needle to the MHA plate. The wells that contained cinnamon bark extract showed growth at the agar plate until the concentration of extract was 2048 µg/mL. Therefore, the MBC could not be obtained. It is assumed that the MBC might be greater than 2048 µg/mL (see Table 4).

Antimicrobial combinations were then tested, and a better interaction between the extract of cinnamon and honey against those bacteria was expected. When two drugs are given simultaneously, interactions in the body that cause pharmacological effect, be they synergistic, addictive, or antagonist effect, can occur. The combination of extract and honey was evaluated from the FICI values for each combination using the microdilution checkerboard method.

The concentration of the individual compounds in combination between the extract and honey, which inhibit bacterial growth, was recorded as the MIC of the individual compounds in the respective combination. The combination of cinnamon bark extract and honey against *P. acnes* and *S. epidermidis* showed an additive effect with an FICI value of 0.625 (see Table 5). This means that the combination effect of cinnamon bark extract and honey was equal to the sum of the effect of each agent alone. The combination of the ethanolic extract of cinnamon bark and honey had potential activity against acne-causing bacteria. In addition, since both agents have a good flavor and texture, there is a promising prospect to develop them as topical treatments against acne-causing bacteria.

## 4. Conclusions

The MICs of the ethanolic extract of cinnamon bark against *P. acnes* and *S. epidermidis* were 256 µg/mL and 1024 µg/mL, respectively. The MICs of honey against *P. acnes* and *S. epidermidis* were 50% (*v*/*v*) and 50% (*v*/*v*), respectively. The combination of extract of cinnamon bark and honey against *P. acnes* and *S. epidermidis* showed an additive activity with a FICI value of 0.625. It is consequently suggested that the extract of cinnamon bark and honey has good potential activity against acne-causing bacteria, and hence can be developed as topical anti-acne preparations.

## Figures and Tables

**Table 1:** Characterization of cinnamon bark extract.

Test	Result
Water content (*v*/*w*)	7.0%
Total ash (*w*/*w*)	6.5%
Ethanol extractable matter (*w*/*w*)	16.0%
Water extractable matter (*w*/*w*)	11.3%
Yield % (*w*/*w*)	15.93%

**Table 2:** Phytochemical screening of crude drug and extract of cinnamon bark.

Compounds	Crude Drug	Extract
Alkaloid	+	+
Flavonoid	+	+
Steroid/Triterpenoid	+	+
Tannin	+	+
Saponin	−	−
Quinone	+	+

Note: (+) Detected. (−) Not detected.

**Table 3:** Antibacterial screening of the ethanolic extract of cinnamon bark, honey, and tetracycline HCl.

Strains	Diameter of Zone of Inhibition (mm)		
	Cinnamon Bark Extract (90%) (*w*/*w*)	Honey (100%) (*v*/*v*)	Tetracycline HCl (250 ppm) (mg/mL)
*P. acnes*	17.2	16.2	18.9
*S. epidermidis*	16.8	16.7	24.8

**Table 4:** Result of MIC and MBC of cinnamon bark extract, honey, and tetracycline HCl.

Strains	Cinnamon Bark Extract (µg/mL)		Honey (%) (*v*/*v*)		Tetracycline HCl (µg/mL)	
	MIC	MBC	MIC	MBC	MIC	MBC
*P. acnes*	256	>2048	50	100	8	8
*S. epidermidis*	1024	>2048	50	100	2	2

MIC: minimum inhibitory concentration; MBC: minimum bactericidal concentration.

**Table 5:** The result of the fractional inhibitory concentration index (FICI) between cinnamon bark extract and honey against tested bacteria.

Strains	Combination Extract + Honey	MIC (µg/mL)		FIC		Outcome
		Alone	Combination	FIC	FICI	
*P. acnes*	Cinnamon extract	256	32	1/8	0.625	additive
	Honey	50%	25%	1/2		
*S. epidermidis*	Cinnamon extract	1024	128	1/8	0.625	additive
	Honey	50%	25%	1/2		
